# The perceptions and experiences of people injured in motor vehicle crashes in a compensation scheme setting: a qualitative study

**DOI:** 10.1186/s12889-015-1739-9

**Published:** 2015-04-25

**Authors:** Darnel Murgatroyd, Keri Lockwood, Belinda Garth, Ian D Cameron

**Affiliations:** John Walsh Centre for Rehabilitation Research, Kolling Institute, The University of Sydney, Sydney, NSW Australia

## Abstract

**Background:**

The evidence that compensation related factors are associated with poor recovery is substantial but these measures are generic and do not consider the complexity of scheme design. The objectives of this study were to understand people’s perceptions and experiences of the claims process after sustaining a compensable injury in a motor vehicle crash (including why people seek legal representation); and to explore ways to assist people following a compensable injury and improve their experience with the claims process.

**Methods:**

A qualitative study in a Compulsory Third Party (CTP) personal injury scheme covering the state of New South Wales (NSW), Australia. A series of five focus groups, with a total of 32 participants who had sustained mild to moderate injuries in a motor vehicle crash, were conducted from May to June 2011 with four to eight attendees in each group. These were audio-recorded and transcribed. The methodology was based on a grounded theory approach using thematic analysis and constant comparison to generate coding categories for themes. Data saturation was reached. Analyst triangulation was used to ensure credibility of the results.

**Results:**

Five primary themes were identified: complexity of the claims process; requirement of legal representation; injury recovery expectations; importance of timely healthcare decision making; and improvements for injury recovery. Some participants struggled, finding the claims process stressful and subsequently sought legal advice; whilst others reported a straight forward recovery, helpful insurer interactions and no legal representation. Most participants were influenced by injury recovery expectations, and timely healthcare decision making. To assist with injury recovery, access to objective information about the claims process using online technology and social media was considered paramount.

**Conclusions:**

Participants had contrasting injury recovery experiences and their perceptions of the claims process differed and were influenced by injury recovery expectations, and timeliness of healthcare decision making. Improvements to the claims process are required, including: simplification or streamlining (possibly using online technology and/or social media to reduce paperwork); and providing access to objective information. There is a need to trial early interventions and new claims management policies that could improve injury recovery and satisfaction with the claims process.

## Background

There is now sufficient evidence that compensation related factors are associated with poor recovery following injury [1-5]. However, it is less obvious which aspects of compensation systems are responsible and there is still much debate [6,7]. Research has shown that changing scheme legislation can contribute positively to people’s recovery by removing specific entitlements (e.g. general damages for pain and suffering), encouraging early claims lodgement, and/or early access to treatment [8,9]. Other factors such as claim duration or legal representation have also been shown to influence recovery [10-14].

Currently, compensation related factors in the literature are defined generically, for example: compensation status, claim type/duration, and legal representation [3-5,11-13]. There is little consideration of scheme design complexity. Although qualitative studies investigating the claims experience following injury have found that the adversarial aspects of scheme design can undermine recovery and hinder return to work [15-18].

Similarly, other emerging evidence indicates that the influence of compensation schemes is multi-factorial with the stressfulness of claiming compensation contributing to increased disability and poor psychological function post injury [19-21]. However, there is also the suggestion that baseline mental health plays a significant role in whether or not people find the claims process stressful [19,20].

Further exploratory research is needed to identify how to positively influence distinct compensation systems so that people’s interactions with insurers lead to improved rather than poorer health post injury; whilst maintaining scheme equity and affordability.

This exploratory study examined the experiences of people who had a compensation claim in a single scheme setting. Specifically, the study sought to answer the following questions:

1. What are people’s perceptions and experiences of the claims process after sustaining a compensable injury in a motor vehicle crash?
2. Why do people seek legal representation?
3. How can people be assisted following a compensable injury and their experience with the claims process improved?

## Methods

### Study population and design

The Motor Accidents Authority (MAA) is the government insurance regulator of the Compulsory Third Party (CTP), personal injury scheme in New South Wales (NSW), Australia. The scheme is privately underwritten and predominantly fault-based providing lump sum compensation for entitlements including economic and non-economic loss, and medical expenses. However, there is also limited access to entitlements (e.g. medical expenses and lost wages) for those at fault. Legal representation can be sought at any stage during a claim.

NSW has a population of just over seven million with 26,753 road casualties recorded in 2010/2011. The propensity to claim during this period was 39% and the MAA received 13,373 new claim notifications [22]. Potential participants for this study were identified via the MAA claims database from March 2010 to February 2011. Criterion sampling was used [23] where inclusion criteria consisted of adults over 18 years with mild to moderate injuries (e.g. soft tissue injuries and simple fractures) following a motor vehicle crash in the last three months. Exclusion criteria were severe traumatic brain injury, spinal cord injury or over seven days hospitalisation.

There were 1518 eligible claims lodged within three months of injury between March and December 2010, with 626 invited to participate in a prospective cohort study investigating predictors for poor recovery at 12 and 24 months post injury. Consent and baseline data were obtained from 417 participants. From this sample of 417, a subset of 315 potential participants was obtained for the focus groups following additional exclusions for inability to communicate in English or attend due to geographical distance of home residence. Given the exploratory nature of this study, focus groups were chosen because of their capacity for open discussion between small groups of people with similar experiences, where interaction between participants can facilitate the clarification of ideas [23].

Potential participants were mailed an invitation letter in May 2011 with selection criteria, aims and practical information about the focus groups. They were contacted by telephone within two weeks and asked if they were interested in contributing to focus groups about their perceptions and experiences of the claims process. If agreeable, they were consented and allocated a group based on their availability to attend on a specific date and location.

Of the 193 contacted (106 non-contactable, 16 disconnected numbers) 147 declined, reasons included lack of interest, personal/work commitments and travel time required. Six to nine attendees were allocated per group to allow for lack of attendance. A participation letter was sent including relevant details and all potential participants received a reminder phone call prior to their group. There was no payment but catering and transport costs were covered. Of the 46 that accepted, 32 attended (five focus groups with a minimum of four and a maximum of eight attendees in each group, see Table 1). The remaining 14 were unable to attend due to other commitments, illness or provided no reason.

**Table 1:** **Focus group attendees (n = 32)**

**Group**	**Attended (yes)**	**Attended (no)**
**1**	4	4
**2**	7	2
**3**	6	3
**4**	7	2
**5**	8	3
**Total**	32	14

The study was approved by the Sydney Local Health Network and the University of Sydney Human Research Ethics Committees.

### Data collection

Socio-demographic and injury data were obtained from the existing population cohort study database. Injuries were coded using the Abbreviated Injury Scale (AIS) (2005) at claim lodgement and reviewed within 12 months of injury [24]. The Injury Severity Score (ISS) is an indicator of potential mortality and is calculated by summing the squares of the three highest AIS scores from different body regions [25]. The AIS ranks injuries to particular body regions on a scale from one to six (six is not survivable). Additional information obtained by telephone included claim settlement, legal representation and return to work (Y/N).

All focus groups were facilitated by DM with assistance from KL for two groups (4, 5). Each session lasted one and half hours to allow sufficient time for discussion. A script introducing confidentiality, group procedure and the MAA’s interest in compensation factors was followed on each occasion. No directive feedback was provided. The groups were recorded and transcribed with consent. The issues explored are shown in Table 2 with their corresponding questions.

**Table 2:** **Investigator questions**

**Topic**	**Investigator questions**
Injury recovery experience	How was your experience following injury?
	• What was your experience with the insurer like?
	• What was your experience with health professionals like?
	• What was your experience with the insurer like?
	What was your experience with a lawyer like (why did you get/not get a lawyer)?
Assistance with injury recovery experience	What has assisted you in your recovery?
	What has hindered you in your recovery?
Improvements to injury recovery experience	What could be improved to help people involved in a motor vehicle crash?

### Data analysis

The qualitative methods were based on a grounded theory approach [26,27]. Thematic analysis of the data was conducted independently by DM and KL with no preconceived categories, that is: open coding was used initially where data were coded if it related to the aims of the study. Patterns and themes emerged from these codes, and the constant comparison method was used to generate and refine categories and sub-categories [26,28]. Analysis was completed when data saturation was reached and no new themes emerged in the final two groups [26]. Agreement was reached between the investigators on all themes. Analyst triangulation was also used with a third investigator (IC) independently reviewing the transcripts followed by a group discussion to ensure credibility of the results and consensus was attained for all themes [28].

## Results

The participant profile is outlined in Table 3. Each participant had lodged a compensation claim between six and 15 months previously (median 10 months). Over half of the attendees had returned to work, and of those not working, six were retired/at home and two were receiving a disability pension.

**Table 3:** **Participant profile in the five focus groups (n = 32)**

**Variable**	**Participant data**
**Age range, mean (years)**	22-79, 47
**Gender (male, female)**	12, 20
**Injury severity score range, median**	1-14, 2
**Time since injury range, median (months)**	6-15, 10
Education*	
Primary school	1
Secondary school	8
Certificate or diploma	12
Bachelor degree or higher	11
**Pre-injury occupation (at time of crash)** ^**†**^	
Managers and professionals	12
Technicians, trades, community and personal service workers	2
Clerical, administrative and sales workers	5
Machinery operators, drivers and labourers	0
Unknown, retired/home duties, disability pension	3, 8, 2
**Returned to work at time of focus group (yes/no)**	19, 13
**Claim settled at time of focus group (yes/no)**	12, 20
**Legally represented (yes/no)**	12, 20

*^†^Measures for occupation and education were from the Australian Standard Classification of Occupations (ASCO), 2^nd^ edition and the Australian Standard Classification of Education (ASCED) 2001.

Primary themes were prevalent throughout all five groups, whereas secondary themes were less common but still relevant to our study objectives (see Table 4). The two main topics from which the themes emerged (the claims/legal process and injury recovery) were seen from opposing viewpoints as illustrated by the quotations. The themes are displayed with a narrative and specific quotations.

**Table 4:** **Primary and secondary themes**

	**Theme**
**Primary**	
	Complexity of the claims process
	Requirement of legal representation
	Injury recovery expectations
	Importance of timely healthcare decision making
	Improvements for injury recovery
**Secondary**	
	Desire for financial compensation
	Lack of trust by insurers
	Medico-legal assessments
	Family and social support

### Primary themes

#### Complexity of the claims process

Many participants considered the claims process was unduly complicated with poor insurer communication and customer service. This was often combined with delayed treatment approvals which affected continuity of care.

“My case manager … she is terrible, she doesn’t return phone calls, she doesn’t return emails, I sent her an email two weeks ago and I’ve heard nothing.” (Group 1)

“You talk with someone on the phone and they say okay, we do that and then you get a letter that refuses it, that’s happened to me twice already, and my case manager has changed three times and every time I have to tell them the whole story.” (Group 2)

“I found the company’s intent and their action quite discrepant… I got the phone calls up front, you know, we’ll help you, we’ll do this… then the next thing I heard about it was… A case of turning up one day [at the physiotherapist] and her saying, you’re not covered anymore… no phone call, how are you going? Just you’re not covered anymore.” (Group 3)

In particular, participants with a psychological or chronic illness, or those from a Non English Speaking Background (NESB) often found the claims process especially difficult to negotiate.

“They refused all the treatment that my doctor requested… I’ve done everything they asked me to do, I even returned to work part-time, I mean I am working two days per week with great difficulty… that’s what keeps me off the depression.” (Group 2)

“I can get quite ill if I get too much physical or psychological stress… [it has] just been so terrible because all I wanted to do was just get better… they just passed me from one person to another, can’t get any sense out of them.” (Group 5)

“My English is bad, my brain doesn’t work, she asks something and I think something else and she took it in the wrong way.” (Group 5)

However, others had the opposite experience. They found insurers helpful, with regular communication and timely approvals for treatment. These participants tended to describe less complicated claims.

“I had a guy helping me out. He asked if there was anything else I needed aside from physio… And he called me up after I finished my treatment, maybe a month or two later to see if I was alright. So overall it was pretty good but it was only a small situation.” (Group 2)

“I had to have an operation… they fast tracked everything… just went out of their way… they’ve approved everything I’ve asked for.” (Group 3)

“A slight injury with whiplash and I only needed five sessions of physiotherapy and no real ongoing problems… The whole process seemed to be fairly simple.” (Group 4)

#### Requirement of legal representation

Participants dissatisfied and frustrated with the claims process and lack of objective information commonly turned to legal advice for support. This was particularly relevant when recovery was thought to be impeded.

“I’m now considering going to a lawyer because my condition is no better… I feel I need to get a lawyer to bat for me because I don’t know the finer points of the legal system.” (Group 2)

“They certainly didn’t give any indication of how the system works, so it was a lot of googling, arguing with them, what happens next… I ended up engaging a lawyer simply because I felt I wasn’t getting any cooperation.” (Group 4)

“I thought I can’t, I’m going to a solicitor because I can’t do it, it’s too much.” (Group 5)

For others the engagement of a lawyer related to entitlements.

“People just said you must get a lawyer, I didn’t know and then when I started researching, oh that’s why you need a lawyer… You get a better outcome… lawyers know what to do, that’s their job they know what to claim for and what not to.” (Group 3)

On the contrary, participants who felt their claims were straightforward were less likely to seek a legal opinion.

“It’s never occurred to me… mine’s too minor I guess.” (Group 2)

“In my case it was quite straightforward, there was no question. I was at a standstill and he just went into the back of me.” (Group 4)

#### Injury recovery expectations

Participants had differing expectations regarding injury recovery, dependent on their personal experiences and beliefs. Some participants perceived their recovery was relatively easy, reporting minimal pain and disability. These participants tended to cope well with the claims process.

“Started getting some pains in my back so went for a few months of physio and that was about it. Sort of cleared up.” (Group 2)

“A slight injury with whiplash… no real ongoing problems since then, just occasional twinges… The whole process seemed to be fairly simple.” (Group 4)

Alternatively, others described their pain in powerful terms linked to poor recovery. They demonstrated high levels of disability intertwined with their pain.

“Didn’t think that it was going to be almost 16 months later that I was sitting here… still in nine out of ten pain; it’s ridiculous.” (Group 1)

“I have such severe whiplash that the nerves that my hands got affected… I feel that my flesh is coming off the bones… I can’t drive very far… I can’t hold the browser [sic computer mouse].” (Group 2)

“Pain is there, like you’re confused and like you feel lonely… other people destroy my life, so I can’t work now, I can’t dance, I can’t swim… and now I can do nothing.” (Group 5)

Participants who struggled with prolonged high levels of pain and/or disability expressed dissatisfaction when they sensed disbelief by health professionals of their injury, and felt vindicated when diagnostic scans displayed evidence of an injury. Others wanted testing and a medical diagnosis to understand their symptoms; believing this was essential to aide recovery and appropriate treatment decisions.

“They [the General Practitioner] referred me to get CT scans which actually showed that there were problems with my spine, so that like legitimised the pain… I knew that something was wrong and it was just frustrating that they [the General Practitioner] didn’t believe me, I found that quite unfair.” (Group 3)

“They [the insurer] didn’t approve for MRI, seven months now… they approved for me to go to do physiotherapy, which is silly because you don’t know what is wrong inside my body… I did this for six months and it didn’t work, my body’s hurting me… I’m using all these drugs.” (Group 5)

#### Importance of timely healthcare decision making

The role of health professionals in timely decision making about treatment was perceived as important, particularly as requests for compensable treatment needed to be approved by the insurer prior to commencement of the service. Lack of consistent decision making between health professionals, coupled with poor care coordination, also confused participants.

“I had one shoulder specialist that just said ‘you’ll be fine love, it’ll fix, it’ll fix.’ Now I’m getting told that I have to have a reconstruction, so I can’t see how he could have said that.” (Group 1)

“I don’t know if I will be able to go back to full-time work… I’ve seen some specialists and no one can tell me if I will get better… the worse for me is not knowing how much longer… what the outcome is going to be.” (Group 2)

Any poorly timed decision making then added tension to the triadic relationship between patient, health professional and insurer.

“I’m still waiting for approval to go and see a podiatrist because my arch has collapsed on my foot.” (Group 1)

“They paid all my expenses up until about three weeks ago and then said, right no more physio, so the physio had a stand up fight with them.” (Group 2)

Conversely, if participants received timely treatment and assistance from knowledgeable and supportive health professionals, particularly to negotiate the claims process, satisfaction with their care was greater.

“She [the General Practitioner] has been really good at helping me sort those issues out… she actually cares about where I am going.” (Group 1)

“He [the physiotherapist] contacted the insurance company and said… ‘how do you want me to do this’… so he actually negotiated… I had double sessions, so it depends how good your physio is.” (Group 1)

“I got recommended… this chiropractor who has worked in the insurance industry for a long time… he knew exactly how to prepare my report, saying this person needs this much rehabilitation… I’ve got a pretty good experience.” (Group 3)

#### Improvements for injury recovery

In a privately underwritten scheme, each insurer separately provides information to the claimant about the claims process. If legally represented, the insurer is required to communicate with the claimant via their lawyer and not directly, which can delay the receipt of information. Many participants suggested the claims process needed to be simplified, and that objective information should be provided from a single source.

“Information should be out there… until you’ve been through it you have no idea what to do and normally when you’re in an accident you’re not really in a fit state to know what you have to do… They [the insurer] don’t give you much… They give you as much detail as they want you to have I suppose.” (Group 3)

“Streamline the process, so you don’t have to deal with multiple people and tell them the same story over and over again.” (Group 4)

“Anybody involved in an accident should be given as much information as possible, straight away, about what the procedures are, as clearly as possible.” (Group 4)

This especially applied for those struggling with the claims process. Recommendations included the use of technology, social media, and a reduction in paperwork.

“Be it Facebook, be it video, whatever, we’ve got the technology now where you can actually speak to somebody, that’s my insurer, I’m actually speaking to somebody real, and I think that would actually assist a lot in getting rid of the stigma.” (Group 5)

“I need to have this operation ASAP, instead I’m being told… forms had to be posted… look at the modern technology we’ve got now, surely you still don’t have to send something in post and wait for something to be signed.” (Group 3)

It was also suggested that information pertaining to injury recovery expectations would be useful.

“Some people get well really quickly, some people don’t. It would be good if there was more objective knowledge about this thing, so that you weren’t just floundering about.” (Group 5)

### Secondary themes

#### Desire for financial compensation

Participants sustained mild to moderate injuries, yet some expressed a desire for financial compensation related to their perceived injury severity and need for treatment.

“I thought no this [claim] is worth it, because I am spending a lot of my own money to help myself get better.” (Group 2)

“I’ve got a condition that you [the insurer] don’t understand and none of us know what’s going to happen in the long run because of this accident. Like I have no idea how you’re going to compensate me for that.” (Group 4)

“Another thing is actually knowing what you’re entitled to, telling people what they’re entitled too, that’s important as well.” (Group 3)

#### Lack of trust by insurers

Some participants perceived they were not believed by insurers, which tended to hinder their recovery and denigrate their relationship with the insurer. Mistrust led some to feel they were being discriminated against, even if the insurer was investigating the claim to meet legislative requirements prior to paying entitlements.

“Sometimes you sort of feel victimised by the insurance… you feel like they’re saying you’re a bludger, you don’t want to go back to work… you’re scamming… I want to go back to work, I want my normal” (Group 1)

“They sent several doctors to question me just like I’m a criminal robbing their money. And just wonder whether I’m telling lies, just a horrible experience.” (Group 2)

#### Medico-legal assessments

The usefulness of medico-legal assessments was also questioned. Assessments could be sought by insurers or plaintiff/defendant lawyers with health professionals to provide an opinion about diagnosis, treatment or care needs. There was particular reference to the number and veracity of assessments when recommendations were made by a health professional paid for by the insurer.

“It’s a conflict of interest… They’re [the insurer’s assessor] going to give an outcome or a report that’s going to be favourable to the insurance company… so I think that is fundamentally flawed, completely dated and has got to go.” (Group 2)

“What he [the lawyer] said on the first day that I met him, tracked through… getting medico-legal reports from just about every healthcare professional I’ve ever seen.” (Group 3)

#### Family and social support

The positive influence of family and social support was noted by a number of participants. Having a strong network was important and enabled participants to feel secure. Although in the presence of pain and disability, this did not always transpire to increased independence.

“I’ve got an absolutely fantastic group of friends and family… he [my friend] drives me around if there’s days… that you’re in absolute rank pain like today, I wouldn’t have driven today… You’ve got to have that support network, if you don’t have it, you’d just, I would disintegrate.” (Group 1)

“In terms of just psychological wellbeing, my family was really supportive because going from being like perfectly healthy to having like a really painful injury is like depressing.” (Group 3)

## Discussion

This study explored people’s perceptions and experiences of the claims process after sustaining a compensable injury in a motor vehicle crash, and examined ways to assist people in a compensable environment within the first year post injury. The results revealed contrasting viewpoints. Some participants, namely those with prolonged recovery and/or higher pain or disability, found the claims process complicated, difficult to negotiate and frequently sought legal advice. Limited injury recovery expectations, and greater pain and/or disability, appeared to influence participants’ relationships with insurers; which was aggravated by inconsistent care from health professionals.

On the contrary, others found the claims process easy to navigate; particularly if coupled with an expeditious recovery, positive and supportive interactions with health professionals and insurers, and effective health service delivery. A key factor for satisfaction with the claims process appeared to be a communicative and empathetic relationship with the insurer. In these situations legal representation was often deemed unnecessary.

### Experiences of the claims process

Our study reiterated previous research whereby people with a straightforward injury recovery, positive relationship with health professionals, and timely communication and approvals from the insurer generally felt satisfied with the claims process and their care from health professionals and insurers [15,18,29]; they also appeared to have less complex needs, pain and/or disability, and shorter treatment duration [17]. Our study also identified the presence of strong family and social support as important for recovery, which is supported in the literature [1,13,30].

Conversely, poor insurer communication, customer service and delayed treatment approvals were identified as key areas of concern. This is similarly reflected in different jurisdictions in the United States, Canada and Australia [15-19,29,31].

Many participants struggled with high initial pain intensity, psychological distress and co-morbidities, putting them at risk of poorer outcomes following injury [2,4,5,11-13,32]. In similar research, lack of understanding about claim requirements, claim delays and medico-legal assessments also impacted negatively on psychological function following injury, predominantly in the presence of poor baseline mental health [19,20]. A dearth of evidence exists indicating what might assist these people to recover in a compensable environment.

While many study participants believed injury recovery depended on a definitive diagnosis, evidence indicates that investigations do not provide a diagnosis predictive of outcome or pain [33-36]. Current guidelines allow 10 working days for an insurer to approve or decline a treatment request [37], and if the insurer delayed or denied investigations this compounded dissatisfaction with the claims process. Comparable studies also found that participants expressed frustration and anger when investigations and/or treatment perceived as relevant were questioned or denied [16,17]. This involvement of a third party payer also complicated the health professional-patient relationship, as reiterated in other studies with similar sentiments of disconnect between the parties; particularly over timeframes and resources for making decisions about investigations, treatment and other benefits [16,18].

The desire for financial compensation expressed by some claimants in our study was connected to perceived injury severity and the need for treatment. This appeared to reflect a perceived entitlement to benefits in a more general sense, and not the psychological entitlement (the sense that one deserves more and is entitled to more than others) reported elsewhere in the literature [38,39].

The lack of trust by insurers was based on perceived unfair insurer conduct particularly for access to treatment. In the NSW motor accidents scheme, access to treatment is on a case-by-case basis taking into account the principles of ‘reasonable and necessary’ which include: benefit to the injured person; appropriateness of the service/provider; relationship to the crash, and cost [40]. With this approach, there is the potential for the insurer and participant to disagree. Existing research also relates these secondary themes to power imbalances and stigmatisation [15,17], and perceived injustice; a multidimensional construct that includes unfairness, irreparability of loss and injury severity [21,41].

Our study also highlighted the potential conflict of interest when insurers pay for health professionals to conduct medico-legal assessments. This questionable value of medico-legal assessments is emerging in the literature and likely to be dependent on scheme design [18,19,31]. In other related research, the secondary themes found in our study were identified as primary themes, possibly because participants in other studies sustained more severe injuries, and/or were predominantly over one year post injury [15,17,31].

### Why people sought legal representation

Frustration with the claims process and a perceived lack of insurer cooperation led some participants to seek legal advice. Although legal representation has been shown across different compensation systems to be associated with poorer outcomes, there is no compelling explanation of the mechanisms involved [5,10,11,13,14]. Our findings suggest seeking legal advice may be influenced by the complexity of the compensation system and perceived slow injury recovery, particularly if recovery was thought to be impeded by the claims process. In the NSW motor accidents scheme, legal representation of full claims is almost 60% within the first 12 months [22]. Discussion amongst legal researchers and participants who expressed an opposing point of view supports these concepts [42,43]. In addition, poor baseline mental health, vulnerability to stress and initial pain intensity may also be drivers of seeking legal representation [4,5,11,13,14,19,20].

### Ways to improve the claims process

The extensive information and paperwork required to access entitlements has been well documented [16,18,19]. Similarly, our study found that access to objective information from the scheme regulator and/or health professionals about the claims process (e.g. entitlements, obligations of both parties, legal representation) and injury recovery was important; yet in need of streamlining. Use of new technology and social media was suggested to simplify the process, break down communication barriers and reduce paperwork requirements. Examples include: YouTube and Apps to explain claims procedures, accessing entitlements including timeframes, and insurer obligations to provide reasons for decisions - particularly declinature; FaceTime and Skype for video conferencing to improve communication and possibly reduce stigmatisation; and Facebook or WhatsApp for information sharing about ways to optimise injury recovery – staying active or when to seek medical advice. This call for assistance has been found across jurisdictions predominantly in schemes with adversarial components such as: legislative requirements for proving causation of injury or receiving benefits for lost wages; timeframes to access to entitlements; and/or a propensity for seeking legal representation [15,17,19,29,44]. A recent rapid review also reported a lack of evidence surrounding the effectiveness of interventions that focussed on information about the claims process [45].

### Study strengths and limitations

Due to the diversity of compensation scheme design, generalisability is limited. Only participants relatively early in the claims cycle with minor to moderate injuries were included. Conversely, contemporary information is provided about perceptions of the claims process and injury recovery experience in this early phase. Much of the literature refers purely to Workers Compensation jurisdictions; this study adds to the few involving road traffic injury schemes [19,29,31]. Lastly, these focus groups were conducted using established methodology which resulted in robust data with across group data saturation [26-28].

### Further research and policy implications

These findings provide an opportunity to understand the complexity of compensation systems and new evidence about which aspects of the claims process could be associated with poor recovery such as: poor insurer communication; delays in decision making about treatment; the triggers for seeking legal representation; and the lack of objective information. Moreover, risk factors for poor prognosis and co-morbidities, especially poor mental health, appear to be aggravated by the claims process. There is a need to trial early interventions and new claims management policies that could improve injury recovery and satisfaction with the claims process.

Such interventions could include: streamlining claim lodgement and treatment requests with online facilities to reduce delays; a comprehensive information package from an independent source such as the scheme regulator; face-to-face communication with participant and insurer in person or online; early identification of risk factors for poor recovery; and subsequent early appropriate treatment referrals. Further consideration should be given to consolidating resources for those at risk and minimising insurer involvement with those recovering well.

Notwithstanding that, further qualitative research is needed with different injury groups, timeframes and compensation schemes to consolidate themes. In addition, the role of having a NESB needs to be explored to understand how to assist this population.

## Conclusions

The findings of this study echo those of other jurisdictions regardless of scheme design. Participants had contrasting injury recovery experiences. Some participants found the claims process stressful and subsequently sought legal advice; whilst others reported a straight forward recovery, helpful insurer interactions and no legal representation. Both groups were influenced by injury recovery expectations and timeliness of healthcare decision making. The triadic relationship between the parties could particularly aggravate health service delivery.

Improvements to the claims process are required including: simplification or streamlining – possibly using online technology and/or social media to reduce paperwork; and providing access to objective information. Addressing some of the negative issues raised by participants could reduce the adversarial nature of the claims process in a compensable setting and alleviate the triggers for seeking legal representation; thereby providing a more optimal environment for injury recovery.
